# Supplementary material for: Prion seeding activity in DNA extractions: implications for laboratory biosafety
Source: Prion. 2026 Jan 29;20(1):1–16. doi: 10.1080/19336896.2026.2619277 (PMC12867400; doi:10.1080/19336896.2026.2619277)
Supplement: Appendix A Table A1.pdf [file KPRN_A_2619277_SM1496.pdf]

				MagAttract DNA		Dneasy DNA		Tissue
Animal ID	Animal's CWD Status (by ELISA or IHC)	Sample ID	Tissue	RT-QuIC Results	[DNA] (ng/uL)	RT-QuIC Results	[DNA] (ng/uL)	RT-QuIC Results
4	-	25	brain	-	232	-	323	-
		26	parotid LN	-	504	-	300	-
5	-	27	brain	-	87	-	18	-
		28	parotid LN	-	164	-	7.7	-
6	-	29	brain	-	302	-	140	-
		30	parotid LN	-	61	-	0.7	-
7	-	32	parotid LN	-	0.2	-	306	-
8	-	33	brain	-	1.1	-	19	-
		34	parotid LN	-	2.7	-	21	-
9	-	35	brain	-	76	-	28	-
		36	parotid LN	-	512	-	590	-
10	-	37	brain	-	21	-	5	-
		38	parotid LN	-	3.6	-	12	-
31	-	3	parotid LN	-	240	-	240	-
		4	brain stem	-	33	-	9.1	-
		15	muscle	-	59	-	0.3	-
32	-	1	RPLN	-	260	-	246	-
		2	brain stem	-	25	-	2.5	-
		13	muscle	-	71	-	7.5	-
313	-	44	cerebrum	-	82	-	4.9	-
840	-	9	RPLN	-	410	-	340	-
		17	brain stem	-	38	-	4.3	-
		22	muscle	-	56	-	0.2	-
