# Supplementary material for: Prion seeding activity in DNA extractions: implications for laboratory biosafety
Source: Prion. 2026 Jan 29;20(1):1–16. doi: 10.1080/19336896.2026.2619277 (PMC12867400; doi:10.1080/19336896.2026.2619277)
Supplement: Appendix A Table A2.pdf [file KPRN_A_2619277_SM1495.pdf]

				MagAttract DNA		Dneasy DNA		Tissue
**Animal ID**	**Animal's CWD Status (by ELISA or IHC)**	**Sample ID**	**Tissue**	**RT-QuIC Results**	**[DNA] (ng/uL)**	**RT-QuIC Results**	**[DNA] (ng/uL)**	**RT-QuIC Results**
287	+	8	brain stem	+	69	+	27	+
		16	muscle	-	55	-	10.6	-
		19	parotid LN	+	286	-	<0.1	+
		55	salivary gland	+	368	+	228	+
		57	submandibular LN	+	45	+	522	+
288	+	39	sciatic nerve	-	60	-	0.8	-
		40	muscle	-	68	-	13	-
289	+	41	sciatic nerve	-	58	-	2.2	-
		42	muscle	-	112	-	7.1	-
306	+	60	ear	+	89	+	226	+
		6	parotid LN	+	202	+	154	+
		12	brain stem	+	32	+	5	-
		23	muscle	-	36	-	60	-
307	+	48	parotid LN	+	266	+	464	+
		49	muscle	-	0.7	-	0.7	-
		82	mesenteric LN	+	0.3	+	2.2	+
		83	salivary gland	+	16	+	118	-
		84	ear	+	164	+	196	+
		85	bone marrow	+	80	-	74	+
		86	popliteal LN	+	4.8	+	5	+
		87	spleen	+	36	+	40	+
		88	rectum	+	32	+	116	+
		89	eye	-	0.6	-	0.2	+
		91	inguanal LN	+	104	+	400	+
		92	prescapular LN	+	20	+	17	+
		93	gastrohepatic LN	+	<0.1	+	0.3	+
		95	tonsil	+	94	+	14	+
		96	third eyelid	+	82	+	466	+
310	+	10	brain stem	+	73	+	5	+
		11	RPLN	+	322	+	490	+
		14	muscle	-	5.7	-	39	-
		99	submandibular LN	+	236	+	1160	+
		100	third eyelid	+	67	+	125	+
		101	parotid LN	+	164	+	744	+
		102	tongue	-	60	-	31	+
		103	ear	+	48	+	290	-
		105	salivary gland	+	5.8	+	108	+
312	+	43	cerebrum	-	103	-	8.5	-
316	+	5	RPLN	+	742	+	408	+
		18	brain stem	-	110	-	11	-
		21	muscle	-	50.4	-	8.6	-
		63	parotid LN	+	23	+	148	+

		64	brain	-	47	+	5.3	+
		65	tongue	+	124	+	158	+
319	+	7	brain Stem	+	41	+	9.2	+
		20	parotid LN	+	118	+	524	+
		24	muscle	-	66	-	0.1	-
		74	third eyelid	+	119	+	137	+
		75	tonsil	+	160	+	240	+
		77	popliteal LN	+	185	+	1620	+
		78	submandibular LN	+	564	+	568	+
		80	brain	+	55	+	0.3	+
		81	salivary gland	+	96	+	154	-
