# Supplementary material for: Prion seeding activity in DNA extractions: implications for laboratory biosafety
Source: Prion. 2026 Jan 29;20(1):1–16. doi: 10.1080/19336896.2026.2619277 (PMC12867400; doi:10.1080/19336896.2026.2619277)
Supplement: Appendix B Table B2.pdf [file KPRN_A_2619277_SM1494.pdf]

Sample ID	Tissue type	ELISA	OD value	IHC	DNA extraction KIT
20RC0080	Obex	POS	NA	POS	QIAcube HT
20RC0081	Obex	POS	0.776	LN POS/ OB NEG	QIAcube HT
20RC0083	Obex	POS	NA	POS	QIAcube HT
20RC0084	Obex	POS	NA	POS	QIAcube HT
20RC0085	Obex	POS	NA	POS	QIAcube HT
20RC0088	Obex	POS	NA	POS	QIAcube HT
20RC0089	Obex	POS	NA	POS	QIAcube HT
20RC0091	Obex	POS	NA	POS	QIAcube HT
22RC0054	Obex	NEG	0.024	NEG	QIAcube HT
22RC2286	Obex	NEG	0.009	NA	QIAcube HT
22RC2360	Obex	NEG	0.014	NA	QIAcube HT
22RC2362	Obex	NEG	0.018	NA	QIAcube HT
22RC2391	Obex	NEG	0.018	NA	QIAcube HT
22RC2415	Obex	NEG	0.023	NA	QIAcube HT
22RC2422	Obex	NEG	0.008	NA	QIAcube HT
22RC2433	Obex	NEG	0.015	NA	QIAcube HT
22RC2434	Obex	NEG	0.014	NA	QIAcube HT
22RC2437	Obex	NEG	0.016	NA	QIAcube HT
22RC2588	Obex	NEG	0.013	NA	QIAcube HT
22RC2589	Obex	NEG	0.013	NA	QIAcube HT
22RC2594	Obex	NEG	0.013	NA	QIAcube HT
22RC2621	Obex	NEG	0.011	NA	QIAcube HT
22RC2648	Obex	NEG	0.004	NA	QIAcube HT
22RC2746	Obex	NEG	0.018	NA	QIAcube HT
22RC2775	Obex	NEG	0.031	NA	QIAcube HT
22RC2808	Obex	NEG	0.011	NA	QIAcube HT
22RC3019	Obex	NEG	0.006	NA	QIAcube HT
22RC3274	Obex	NEG	0.008	NA	QIAcube HT
22RC3328	Obex	POS	NA	POS	QIAcube HT
22RC3331	Obex	POS	NA	POS	QIAcube HT
22RC3335	Obex	POS	NA	POS	QIAcube HT
22RC3340	Obex	POS	NA	LN POS/ OB NEG	QIAcube HT
22RC3346	Obex	POS	NA	POS	QIAcube HT
22RC3348	Obex	POS	0.053	POS	QIAcube HT
22RC3351	Obex	POS	NA	POS	QIAcube HT
22RC3355	Obex	POS	NA	POS	QIAcube HT
22RC3362	Obex	POS	NA	LN POS/ OB NEG	DNeasy
22RC3580	Obex	POS	NA	POS	QIAcube HT

22RC3586	Obex	POS	NA	POS	QIAcube HT
22RC3590	Obex	POS	NA	POS	QIAcube HT
22RC3594	Obex	POS	NA	POS	QIAcube HT
22RC3600	Obex	POS	NA	POS	QIAcube HT
22RC3601	Obex	POS	NA	POS	QIAcube HT
22RC3604	Obex	POS	NA	POS	QIAcube HT
22RC3605	Obex	POS	NA	LN POS/ OB NEG	QIAcube HT
22RC3611	Obex	POS	NA	POS	QIAcube HT
22RC3612	Obex	POS	NA	POS	QIAcube HT
22RC3622	Obex	POS	NA	POS	QIAcube HT
24RC0005	RPLN	NEG	0.02	NA	QIAcube HT
24RC0006	RPLN	NEG	0.013	NA	QIAcube HT
24RC0007	RPLN	NEG	0.015	NA	QIAcube HT
24RC0008	RPLN	NEG	0.013	NA	QIAcube HT
24RC0009	RPLN	NEG	0.014	NA	QIAcube HT
24RC0010	RPLN	NEG	0.012	NA	QIAcube HT
24RC0011	RPLN	NEG	0.015	NA	QIAcube HT
24RC0012	RPLN	NEG	0.017	NA	QIAcube HT
24RC0013	RPLN	NEG	0.022	NA	QIAcube HT
24RC0014	RPLN	NEG	0.014	NA	QIAcube HT
24RC0015	RPLN	NEG	0.011	NA	QIAcube HT
24RC0016	RPLN	NEG	0.005	NA	QIAcube HT
24RC0200	RPLN	POS	NA	POS	QIAcube HT
24RC0201	RPLN	POS	NA	POS	QIAcube HT
24RC0202	RPLN	POS	NA	POS	QIAcube HT
24RC0204	RPLN	POS	NA	POS	QIAcube HT
24RC0205	RPLN	POS	NA	POS	QIAcube HT
24RC0206	RPLN	POS	NA	POS	QIAcube HT
24RC0207	RPLN	POS	NA	POS	QIAcube HT
24RC0208	RPLN	POS	NA	POS	QIAcube HT
24RC0210	RPLN	POS	NA	POS	QIAcube HT
24RC0211	RPLN	POS	NA	POS	QIAcube HT
24RC0212	RPLN	POS	NA	POS	QIAcube HT
24RC0213	RPLN	POS	NA	POS	QIAcube HT
24RC0214	RPLN	POS	NA	POS	QIAcube HT
24RC0215	RPLN	POS	NA	POS	QIAcube HT
24RC0216	RPLN	POS	NA	POS	QIAcube HT
24RC0227	RPLN	POS	3.5	POS	DNeasy
24RC0278	RPLN	POS	NA	POS	QIAcube HT
24RC0279	RPLN	POS	NA	POS	QIAcube HT
24RC0280	RPLN	POS	NA	POS	QIAcube HT
24RC0281	RPLN	POS	NA	POS	QIAcube HT

24RC0283	RPLN	POS	NA	POS	QIAcube HT
24RC0289	RPLN	POS	NA	POS	QIAcube HT
24RC0339	RPLN	POS	NA	POS	QIAcube HT
24RC0342	RPLN	POS	NA	POS	QIAcube HT
24RC0358	RPLN	POS	NA	POS	DNeasy
24RC0017	RPLN	NEG	NA	NA	QIAcube HT
24RC0019	RPLN	NEG	NA	NA	QIAcube HT
24RC0020	RPLN	NEG	NA	NA	QIAcube HT
