# Supplementary material for: Prion seeding activity in DNA extractions: implications for laboratory biosafety
Source: Prion. 2026 Jan 29;20(1):1–16. doi: 10.1080/19336896.2026.2619277 (PMC12867400; doi:10.1080/19336896.2026.2619277)
Supplement: Gresch et al_SupplementalMaterials.docx [file KPRN_A_2619277_SM1493.docx]

**Appendix A**

MNPRO Supplemental Data

**Figure A1.** MNPRO RT-QuIC results for the evaluation of potential DNA inhibitory effects on RT-QuIC performance. Negative DNA elutes (260 ng/µL) were spiked with PrP^Sc^ positive control across a five-point dilutions series. Top panel: Rate of Amyloid Formation, middle panel: Maxpoint Ratio, bottom panel: Max Slope.

**Table A1.** MNPRO RT-QuIC results for tissue and both types of DNA eluates in all CWD-negative WTD samples used in these experiments.

**Table A2.** MNPRO RT-QuIC results for tissue and both types of DNA eluates in all MNPRO CWD-positive WTD samples used in these experiments.

**Table A3.** MNPRO RT-QuIC results for tissue and DNA eluates for all MNPRO Syrian hamster (*Mesocricetus auratus*) samples used in these experiments.

**Table A4.** MNPRO RT-QuIC results for endpoint titration experiments. NT = not tested. Sample IDs here correspond with the Sample IDs shown in Table A2.

**Appendix B**

CFIA Supplemental Data

**Table B1. Concentration of DNA extracted from control tissue homogenates^1^**

^1^Tissue homogenates were mixtures of CWD+ and CWD- tissue homogenate in various ratios, each containing 0.01% (w/v) of tissue specimens. DNA was extracted from the tissue homogenates using the DNeasy Blood & Tissue kit (Qiagen) and quantified using the Quant-iT kit (Invitrogen).

**Table B2**. CFIA sample information

**Table B3.** RT-QuIC data for DNA samples

**Figure B1**. Correlation between the concentration and cycle threshold in DNA samples
