# Supplementary material for: Prion seeding activity in DNA extractions: implications for laboratory biosafety
Source: Prion. 2026 Jan 29;20(1):1–16. doi: 10.1080/19336896.2026.2619277 (PMC12867400; doi:10.1080/19336896.2026.2619277)
Supplement: Appendix A Table A4.pdf [file KPRN_A_2619277_SM1492.pdf]

Sample ID	DNA extraction kit	Animal CWD status (by ELISA or IHC)	DNA status	DNA concentration (ng/uL)	Dilution	Tissue RT-QuIC Result	DNA RT-QuIC Result	Tissue type
82	DNeasy	+	+	2.2	10^{-1}	NT	+	Mesenteric lymph node
					10^{-2}	NT	+	
					10^{-3}	+	-	
					10^{-4}	+	-	
					10^{-5}	+	-	
					10^{-6}	-	NT	
					10^{-7}	-	NT	
86	DNeasy	+	+	5	10^{-1}	NT	-	Popliteal lymph node
					10^{-2}	NT	-	
					10^{-3}	+	-	
					10^{-4}	+	-	
					10^{-5}	+	-	
					10^{-6}	+	NT	
					10^{-7}	-	NT	
92	DNeasy	+	+	17	10^{-1}	NT	+	Prescapular lymph node
					10^{-2}	NT	-	
					10^{-3}	+	-	
					10^{-4}	+	-	
					10^{-5}	+	-	
					10^{-6}	+	NT	
					10^{-7}	-	NT	
					10^{-8}	-	NT	
7	MagAttract	+	+	41	10^{-1}	NT	+	Brain stem
					10^{-2}	NT	-	
					10^{-3}	+	-	
					10^{-4}	+	-	
					10^{-5}	+	-	
					10^{-6}	-	NT	
					10^{-7}	-	NT	
74	MagAttract	+	+	119	10^{-1}	NT	+	Third eyelid
					10^{-2}	NT	+	
					10^{-3}	+	-	
					10^{-4}	+	-	
					10^{-5}	+	-	
					10^{-6}	+	NT	
					10^{-7}	-	NT	
					10^{-8}	-	NT	
6	MagAttract	+	+	202	10^{-1}	NT	+	Parotid
					10^{-2}	NT	+	
					10^{-3}	+	+	
					10^{-4}	+	-	
					10^{-5}	+	-	
					10^{-6}	+	NT	
					10^{-7}	-	NT	

99	MagAttract	+	+	236	10^{-1}	NT	+	Submandibular lymph node
					10^{-2}	NT	-	
					10^{-3}	+	+	
					10^{-4}	+	-	
					10^{-5}	+	-	
					10^{-6}	+	NT	
					10^{-7}	-	NT	
20	DNeasy	+	+	524	10^{-1}	NT	+	Parotid
					10^{-2}	NT	+	
					10^{-3}	+	+	
					10^{-4}	+	-	
					10^{-5}	+	-	
					10^{-6}	+	NT	
					10^{-7}	+	NT	
					10^{-8}	-	NT	
99	DNeasy	+	+	1,160	10^{-1}	NT	+	Submandibular lymph node
					10^{-2}	NT	+	
					10^{-3}	+	+	
					10^{-4}	+	+	
					10^{-5}	+	-	
					10^{-6}	+	NT	
					10^{-7}	-	NT	
43	MagAttract	+	-	103	10^{-1}	NT	-	Cerebrum
					10^{-2}	NT	-	
					10^{-3}	-	-	
					10^{-4}	-	-	
					10^{-5}	-	-	
					10^{-6}	-	NT	
					10^{-7}	-	NT	
