# Supplementary material for: Prion seeding activity in DNA extractions: implications for laboratory biosafety
Source: Prion. 2026 Jan 29;20(1):1–16. doi: 10.1080/19336896.2026.2619277 (PMC12867400; doi:10.1080/19336896.2026.2619277)
Supplement: Appendix A_Figure A1 spiked DNA.pdf [file KPRN_A_2619277_SM1491.pdf]

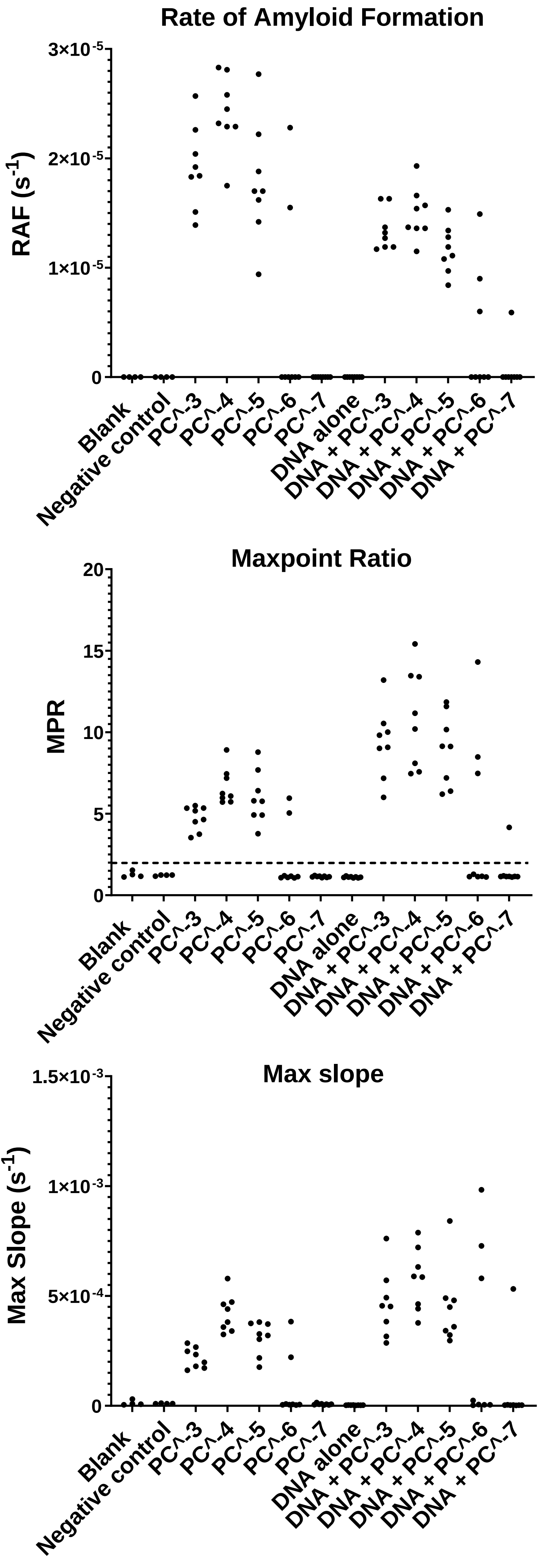

Rate of Amyloid Formation
RAF (s-1)
3×10-5
2×10-5
1×10-5
0
Blank
Negative control
PC^-3
PC^-4
PC^-5
PC^-6
PC^-7
DNA alone
DNA + PC^-3
DNA + PC^-4
DNA + PC^-5
DNA + PC^-6
DNA + PC^-7
Maxpoint Ratio
MPR
20
15
10
5
0
Max slope
Max Slope (s-1)
1.5×10-3
1×10-3
5×10-4
0
