# Supplementary figures and images for: Prion seeding activity in DNA extractions: implications for laboratory biosafety
Source: Prion. 2026 Jan 29;20(1):1–16. doi: 10.1080/19336896.2026.2619277 (PMC12867400; doi:10.1080/19336896.2026.2619277)

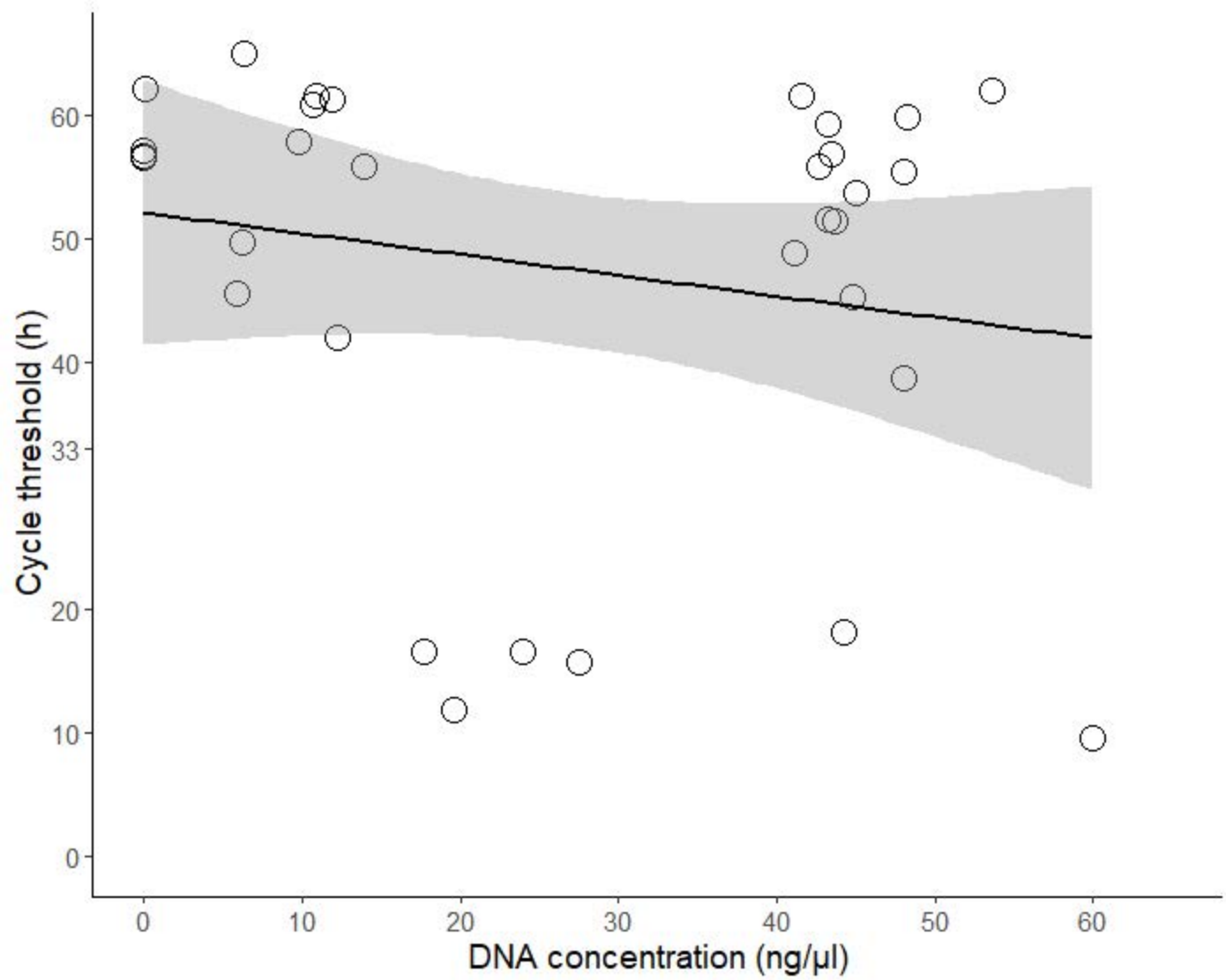

Supplement: Appendix B Figure B1.pdf [file KPRN_A_2619277_SM1490.pdf]
