# Supplementary material for: Prion seeding activity in DNA extractions: implications for laboratory biosafety
Source: Prion. 2026 Jan 29;20(1):1–16. doi: 10.1080/19336896.2026.2619277 (PMC12867400; doi:10.1080/19336896.2026.2619277)
Supplement: Appendix A Table A3.pdf [file KPRN_A_2619277_SM1489.pdf]

		DNeasy DNA Extraction		Tissue
**Sample ID**	**Experimental Status**	**RT-QuIC Results**	**[DNA] (ng/uL)**	**RT-QuIC Results**
HB3	control (negative)	-	7.8	-
HB6	control (negative)	-	7.6	-
HB8	control (negative)	-	4.9	-
HB9	control (negative)	-	4	-
HB11	control (negative)	-	12	-
HB13	control (negative)	-	4.6	-
HB15	control (negative)	-	6.3	-
HB16	control (negative)	-	7.4	-
HB17	control (negative)	-	8.2	-
HB4	80 d post inoculation	+	5.4	+
HB5	80 d post inoculation	+	4.6	+
HB10	120 d post inoculation	+	9.9	+
HB12	120 d post inoculation	+	9.1	+
HB14	120 d post inoculation	+	6.1	+
HB2	160 d post inoculation	+	11	+
HB7	160 d post inoculation	+	8.6	+
HB18	160 d post inoculation	+	8.3	+
