# Supplementary material for: Prion seeding activity in DNA extractions: implications for laboratory biosafety
Source: Prion. 2026 Jan 29;20(1):1–16. doi: 10.1080/19336896.2026.2619277 (PMC12867400; doi:10.1080/19336896.2026.2619277)
Supplement: Appendix B Table B3.pdf [file KPRN_A_2619277_SM1488.pdf]

Sample name	# RT-QuIC replicates	# POS replicates (CT < 30h)	Cycle Threshold (CT) for individual replicate							
20RC0080	4	4/4	11.61	13.24	11.45	10.25				
20RC0081	8	0/8	65	65	65	55.07	56.12	65	46.28	65
20RC0083	8	4/8	8.91	65	11.65	11.61	43.39	69.4	65	11.03
20RC0084	8	7/8	65	8.24	9.24	14.44	7.61	6.76	5.21	6.09
20RC0085	4	4/4	8.35	7.35	13.25	9.7				
20RC0088	8	1/8	31.25	47.61	23.14	65	32.4	77.92	65	65
20RC0089	8	2/8	43.42	8.23	11.89	57.64	65	48.6	65	65
20RC0091	8	1/8	65	65	16.15	30.26	65	64.28	75.75	65
22RC0054	4	0/4	65	65	65	65				
22RC2286	4	0/4	65	65	65	65				
22RC2360	4	0/4	65	65	65	65				
22RC2362	4	0/4	65	65	65	65				
22RC2391	4	0/4	65	65	65	65				
22RC2415	4	0/4	65	65	65	65				
22RC2422	4	0/4	65	65	65	65				
22RC2433	4	0/4	65	65	65	65				
22RC2434	4	0/4	65	65	65	65				
22RC2437	4	0/4	65	65	65	40.22				
22RC2588	4	0/4	65	65	65	65				
22RC2589	8	0/8	65	65	65	40.6	45.6	41.61	69.67	65
22RC2594	8	4/8	65	42.45	22.35	65	21.69	12.45	13.49	65
22RC2621	8	2/8	65	5.98	31.29	65.56	65	25.38	30.65	65
22RC2648	8	0/8	65	62.45	53.35	68.3	65	65	65	65
22RC2746	8	1/8	65	65	65	38.26	23.49	41.42	65	65
22RC2775	4	0/4	65	65	65	65				
22RC2808	8	2/8	19.21	65	27.05	65	78.55	31.13	65	65
22RC3019	8	2/8	34.62	65	65	34.45	29.36	28.51	36.38	41.61
22RC3274	8	2/8	65	65	43.18	65	19.25	26.69	31.27	35.55
22RC3328	8	2/8	65	65	42.65	31.7	65	9.72	41.49	9.69
22RC3331	8	6/8	24.39	21.65	52.42	65	11.46	18.29	11.97	14.24
22RC3335	8	7/8	16.72	21.73	65	16.63	13.31	10.15	13.09	10.23
22RC3340	8	4/8	68.47	35.27	65	17.11	19.26	49.47	24.3	19.06
22RC3346	8	5/8	23.47	28.45	65	43.17	3.72	6.19	37.7	6.18
22RC3348	4	4/4	7.68	18.01	18.59	8.83				
22RC3351	4	4/4	9.2	17.27	12.4	8.95				
22RC3355	4	4/4	6.83	13.76	14.71	10.44				
22RC3362	4	4/4	8.74	16.25	11.52	12.55				
22RC3580	8	2/8	42.07	65	65	40.37	20.26	8.95	65	65
22RC3586	8	2/8	41.42	48.74	65	65	19.26	9.54	66.32	65
22RC3590	4	0/4	65	65	65	65				
22RC3594	4	0/4	65	65	65	65				
22RC3600	8	6/8	9.32	65	19.31	18.83	9.96	8.14	65	16.54
22RC3601	4	4/4	9.29	14.34	17.28	18.66				
22RC3604	4	4/4	9.74	15.62	13.49	14.52				
22RC3605	8	6/8	31.69	14.24	12.45	9.24	65	21.3	19.59	22.43
22RC3611	8	6/8	65	33.18	17.28	19.9	14.25	8.9	7.33	25.47
22RC3612	8	4/8	65	16.25	27.76	31.27	37.51	12.24	65	8.07
22RC3622	8	6/8	65	65	13.44	14.55	9.08	5.81	14.08	8.72
24RC0005	4	0/4	65	65	65	65				

24RC0006	8	1/8	65	65	27.64	65	65	78.43	65	65
24RC0007	8	0/8	65	38.02	65	65	65	65	65	65
24RC0008	4	0/4	65	65	65	65				
24RC0009	4	0/4	65	65	65	65				
24RC0010	4	0/4	65	65	65	65				
24RC0011	8	2/8	65	22.32	14.71	32.55	65	65	65	65
24RC0012	8	3/8	22.32	29.54	65	65	65	31.5	9.36	42.26
24RC0013	8	3/8	26.29	29.35	36.19	20.25	65	65	65	65
24RC0014	8	2/8	65	65	35.25	65	29.43	75.75	20.27	41.69
24RC0015	4	0/4	65	65	65	65				
24RC0016	8	4/8	24.57	65	19.31	38.33	23.21	49.47	26.51	48.34
24RC0200	4	4/4	9.22	16.42	13.44	15.15				
24RC0201	4	4/4	13.05	17.58	20.25	23.08				
24RC0202	8	2/8	17.9	38.69	15.74	34.55	65	65	47.41	65
24RC0204	8	4/8	24.28	13.82	16.26	41.67	16.14	65	65	65
24RC0205	8	5/8	14.67	14.27	27.03	41.65	17.31	17.45	65	65
24RC0206	8	4/8	43.49	39.46	21.17	28.76	18.26	65	65	22.31
24RC0207	8	3/8	31.45	32.7	16.87	65	22.03	20.51	45.75	65
24RC0208	4	0/4	65	65	65	65				
24RC0210	4	4/4	8.23	13.23	11.2	7.13				
24RC0211	4	4/4	10.23	6.75	8.59	7.13				
24RC0212	8	4/8	9.15	28.37	20.37	12.57	39.42	65	65	65
24RC0213	4	4/4	8.72	6.58	7.31	8.74				
24RC0214	8	4/8	19.28	16.39	65	65	25.26	65	38.72	15.2
24RC0215	4	4/4	13.51	14.24	12.22	10.48				
24RC0216	8	1/8	65	65	65	20.4	46.62	57.82	65	68.3
24RC0227	8	5/8	9.65	65	15.17	65	3.36	7.22	11.19	65
24RC0278	8	5/8	13.57	65	19.52	44.44	14.74	45.25	22.85	12.31
24RC0279	8	5/8	14.21	23.27	10.25	65	37.49	12.63	38.59	23.28
24RC0280	8	5/8	13.33	11.17	29.35	19.43	65	44.28	10.91	65
24RC0281	4	4/8	14.24	17.87	12.31	12.27				
24RC0283	8	6/8	16.47	14.24	29.27	65	10.28	21.61	14.59	67.29
24RC0289	8	8/8	10.19	13.23	21.54	25.4	14.08	10.21	28.66	19.1
24RC0339	8	7/8	23.49	14.33	33.4	25.38	12.48	17.56	19.27	18.28
24RC0342	4	4/4	6.72	13.22	17.7	7.13				
24RC0358	4	0/4	65	65	65	65				
24RC0017	8	0/8	65	65	65	65	65	65	65	65
24RC0019	8	0/8	65	65	65	65	65	65	65	65
24RC0020	8	2/8	65	28.5	65	29.27	65	65	65	65
